# Anion Inhibition Profile of the β-Carbonic Anhydrase from the Opportunist Pathogenic Fungus *Malassezia restricta* Involved in Dandruff and Seborrheic Dermatitis

**DOI:** 10.3390/metabo9070147

**Published:** 2019-07-18

**Authors:** Sonia Del Prete, Andrea Angeli, Cynthia Ghobril, Julien Hitce, Cécile Clavaud, Xavier Marat, Claudiu T. Supuran, Clemente Capasso

**Affiliations:** 1 Istituto di Bioscienze e Biorisorse, CNR, Via Pietro Castellino 111, I-80131 Napoli, Italy; 2 Sezione di Scienze Farmaceutiche e Nutraceutiche, Dipartimento Neurofarba, Università degli Studi di Firenze, Via U. Schiff 6, I-50019 Sesto Fiorentino, Florence, Italy; 3 L’Oréal Research and Innovation, 93601 Aulnay-sous-Bois, France

**Keywords:** carbonic anhydrases, metalloenzymes, anions, CA inhibitors, *Malassezia restricta*, Malassezia globosa, dandruff, seborrheic dermatitis

## Abstract

Carbonic anhydrases (CAs, EC 4.2.1.1) are ubiquitous metalloenzymes, which catalyze the crucial physiological CO_2_ hydration/dehydration reaction (CO_2_ + H_2_O ⇌ HCO_3_^−^ + H^+^) balancing the equilibrium between CO_2_, H_2_CO_3_, HCO_3_^−^ and CO_3_^2−^. It has been demonstrated that their selective inhibition alters the equilibrium of the metabolites above affecting the biosynthesis and energy metabolism of the organism. In this context, our interest has been focalized on the fungus *Malassezia restricta*, which may trigger dandruff and seborrheic dermatitis altering the complex bacterial and fungal equilibrium of the human scalp. We investigated a rather large number of inorganic metal-complexing anions (a well-known class of CA inhibitors) for their interaction with the β-CA (MreCA) encoded by the *M. restricta* genome. The results were compared with those obtained for the two human α-CA isoforms (hCAI and hCAII) and the β-CA from *Malassezia globosa*. The most effective MreCA inhibitors were diethyldithiocarbamate, sulfamide, phenyl arsenic acid, stannate, tellurate, tetraborate, selenocyanate, trithiocarbonate, and bicarbonate. The different K_I_ values obtained for the four proteins investigated might be attributed to the architectural features of their catalytic site. The anion inhibition profile is essential for better understanding the inhibition/catalytic mechanisms of these enzymes and for designing novel types of inhibitors, which may have clinical applications for the management of dandruff and seborrheic dermatitis.

## 1. Introduction

In recent years, the pharmaceutical industry has focused its attention on inorganic drugs in addition to organic compounds [1,2]. This interest has been determined primarily because inorganic compounds are efficiently delivered in the body, in part because they are small molecules/ions, or they exploit oxidation and ligand substitution reactions [1,2]. Some of the well-known inorganic drugs incorporate metal ions, which are used in a variety of human disease treatments, such as silver sulphadiazine (antibacterial); auranofin (antiarthritic); sodium bromide (sedative); mercurochrome (antiseptic); lithium carbonate (anti-depressive); bismuth derivatives (anti-acid); cisplatin and carboplatin (anticancer); and zinc pyrithione and selenium sulfide (anti-dandruff agents) [3,4,5,6]. They are used in modern medicine to control cancer, infections, diabetes, neurological and cardiovascular problems, ulcers, and anti-inflammatory processes [3]. These small inorganic molecules can bind: (i) DNA to influence the protein expression level; (ii) membrane lipids for altering the integrity of the membrane; or (iii) proteins, in order to inhibit their functions [7]. For example, silver(I) ions and most silver compounds are toxic for bacteria, algae, and fungi in vitro [8]. The antibacterial action of the silver ions is due to their ability to irreversibly damage critical enzymes, which are localized in the cell membranes of pathogens [8,9]. In this context, recently small molecules belonging to anions, such as inorganic metal-complexing anions, have been extensively investigated as inhibitors of bacterial, fungal and protozoan metalloenzymes, such as carbonic anhydrases (CAs, EC 4.2.1.1) [10,11,12,13]. CAs are brand-named into eight genetically distinct families (or classes): α, β, γ, δ, ζ, η, θ, and ι [14,15,16,17,18,19]. Since CAs catalyze the crucial physiological CO_2_ hydration/dehydration reaction (CO_2_ + H_2_O ⇌ HCO_3_^−^ + H^+^) [14,18,20,21,22,23,24,25], balancing the equilibrium between dissolved inorganic carbon dioxide (CO_2_), carbonic acid (H_2_CO_3_), bicarbonate (HCO_3_^−^) and carbonate (CO_3_^2−^) [26,27,28,29], their selective inhibition alters the equilibrium of the aforementioned metabolites affecting the biosynthesis and energy metabolism of the organism [30]. The metal-complexing anions, among the several classes of CA inhibitors (CAIs) (e.g., sulfonamides and their isosteres, dithiocarbamates, xanthates, phenols, polyamines, thioxocoumarins, sulfocoyumarins, and coumarins), bind the Zn(II) ion of the enzyme either in a tetrahedral geometry or as trigonal–bipyramidal adducts of the metal ion [12]. CAs are abundant in fungi and yeasts and have a crucial role in CO_2_-sensing in fungi and the regulation of their sexual development and virulence [12]. Generally, fungal genome encodes for one β-CA, even if multiple copies of β-and α-CAs were reported for some fungi [12]. Fascinating is the fact that fungi belonging to the genus *Malassezia* are involved in dandruff and seborrheic dermatitis physiopathology [31]. For example, the β-CA (MgCA) encoded by the genome of *Malassezia globosa* represent an attractive druggable target since the main CA inhibitors (CAIs) can affect the in vivo and in animal model growth of the microorganism. Recently, an extensive study concerning the inhibition profile of MgCA has been reported using CAIs, such as sulfonamides, anions, a series of 6-substituted benzoxaboroles, monothiocarbamates, dithiocarbamates, famotidine, natural polyphenols, and phenols. The results identified efficient and selective inhibitors [32,33,34,35,36,37,38]. Recently, it has been demonstrated that *Malassezia restricta* represents the larger proportion of *Malassezia* sp. on the scalp in various populations and may also trigger dandruff and seborrheic dermatitis, in conjunction with the complex bacterial and fungal equilibrium of the human scalp [39,40,41,42]. The β-CA encoded by the genome of *M. restricta* (MreCA) is a 230 amino acid residues protein, which showed high catalytic activity for the hydration of CO_2_ to bicarbonate and protons, with a k_cat_ of 1.06 × 10^6^ s^−1^ and k_cat_/K_M_ of 1.07 × 10^8^ M^−1^ s^−1^ that is also sensitive to inhibition by the sulfonamide acetazolamide [31]. In the present paper, a range of inorganic/organic anions and other small molecules known as CAIs are investigated for their interaction with MreCA. The results are compared with those reported for the two human α-CA isoforms (hCA I and hCA II) and the β-CA from *Malassezia globosa* in order to better understand the inhibition/catalytic mechanisms of these enzymes fundamental for many physiologic processes, and for designing novel types of inhibitors, which may have clinical applications for the management of dandruff and seborrheic dermatitis. Here, we show that MreCA exhibits a unique anion inhibition profile.

## 2. Results and Discussion

**Figure 1:**
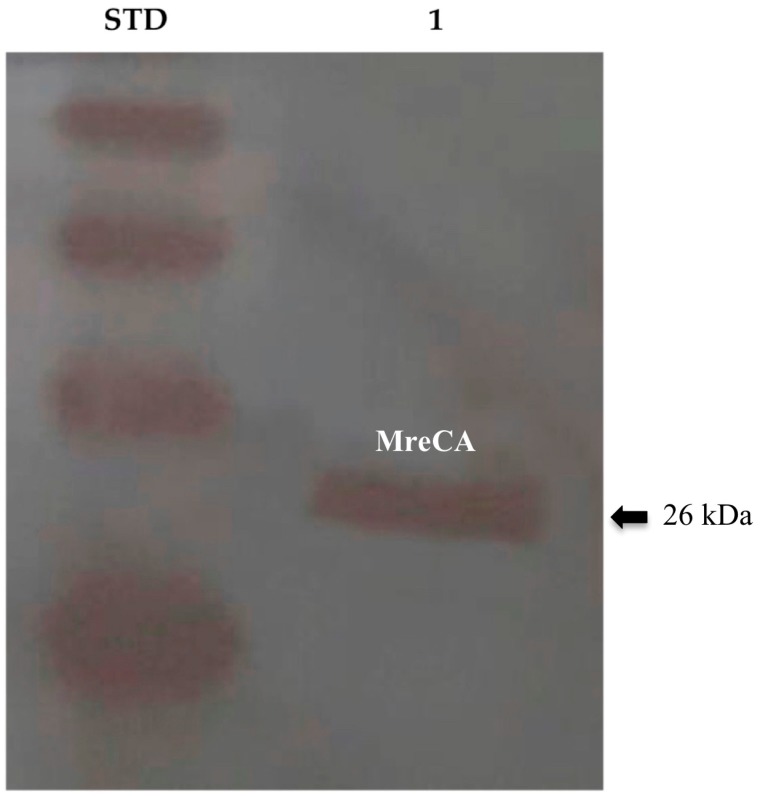
Western blot performed on the purified recombinant MreCA. Legend: Lane Std, MagicMark XP Western Protein Standard, Molecular Weight (M.W.) starting from the top: 50 kDa, 40 kDa, 30 kDa, and 20 kDa, each of which contains an IgG binding site; lane 1, purified MreCA. Bands were identified using the anti-His-Tag antibody.

CAs have a crucial role in fungal biology since they sustain the bicarbonate-dependent carboxylation reactions, are necessary for the sexual reproduction in basidiomycetes and filamentous ascomycetes, and, are involved in CO_2_ sensing by producing the bicarbonate for the activation of adenylyl cyclase (cAMP), which regulates the fungal capsule biosynthesis or filamentation [43,44,45]. Thus, the study of the β-CA from *M. restricta* will be fundamental for a better comprehension of the biological functions of these enzymes, which can interfere with the life cycle of the fungus. In the present study, the recombinant β-CA (MreCA) encoded in the genome of the fungus *M. restricta* was prepared with a tail of six histidines at the C-terminus as reported previously [31]. The purified recombinant MreCA was detected at the molecular weight of about 26.0 kDa, which is the molecular mass of the fusion protein (His-tag + MreCA) (Figure 1).

Additionally, the stopped-flow technique confirmed that the present enzyme showed a high conversion of CO_2_ molecules to bicarbonate per second (k_cat_ =1.06 × 106 s^−1^) and was efficiently inhibited by the classical sulfonamide inhibitor acetazolamide (K_I_ of 50.7 nM) [31]. Intriguingly, MreCA and its homologous enzyme (MgCA) from *M. globosa* are phylogenetically grouped with the β-CA encoded by the genome of *Ustilago maydis*, a pathogenic fungus responsible for the plant disease known as corn smut [46].

**Figure 2:**
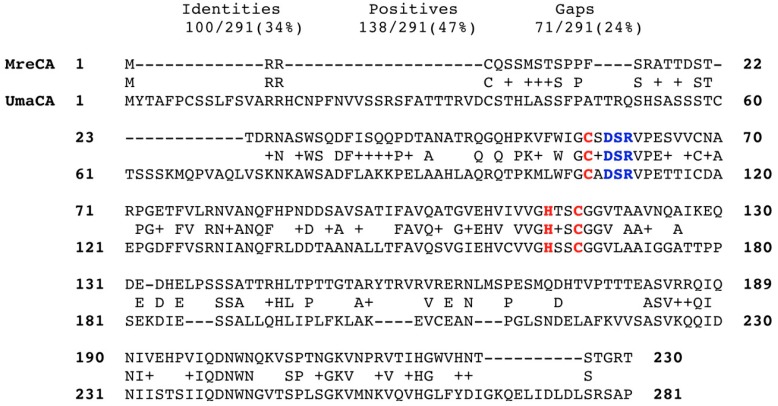
Sequence alignment of the selected β-CAs (MreCA and UmaCA). Zinc ligands are indicated in red; amino acids involved in the enzyme catalytic cycle are indicated in blue. The alignment was performed with the program Blast Global alignment. Legend: MreCA, *Malassezia restricta* β-CA; UmaCA, *Ustilago maydis* β-CA; + symbol, indicates the residues with the same biochemical properties; - symbol, represents the gaps between the amino acid residues.

The MreCA amino acid sequence was aligned with that of the β-CA (acronym UmaCA) from *U. maydis* (Figure 1). The two sequences (MreCA = 230 aa and MgCA= 281 aa) showed that the residues involved in the Zn(II) coordination are all conserved similar to the other β-CAs described in the literature (see Figure 2). Indeed, 100 identical amino acid residues and 138 residues with similar chemical properties (positive residues) have been detected in the sequence analysis of their primary structures (Figure 1). Thus, the identification of inhibitors that could interfere with the life cycle of *M. restricta* might constitute a unique strategy for fighting dandruff and seborrheic dermatitis. Moreover, the discovery of novel molecules targeting and inhibiting the fungal β-CA might offer the possibility to face out the smut of the maize caused by *U. maydis*.

In this context, we investigated a rather large number of inorganic metal-complexing anions for their interaction with MreCA. The inorganic anions represent a well-known class of CA inhibitors (CAIs), due to their affinity for metal ions in solution or when bound within metalloenzyme active sites [47]. The anion inhibition profile of MreCA was compared with those obtained for the human α-CAs (hCA I and hCA II) and the other fungal β-CA (MgCA), which have been previously investigated [33]. The results are shown in Table 1 and the following should be noted:

(i) Anions, which generally complex many cations, including Zn(II), such as fluoride, chloride, bromide, cyanate, thiocyanate, cyanide, nitrite, carbonate, bisulfite, sulfate, hydrogen sulfate, pyrophosphate, divanadate, perrhenate, peroxydisulfate, iminodisulfite, and fluorosulfonate, did not show any inhibitory action against MreCA (K_I_ > 50 mM). It is interesting to note that MreCA has a very intriguingly inhibition pattern versus these anions when compared with its ortholog MgCA, or the two human isoforms (hCA I and hCA II), MrCA being less or not inhibited by most of the anions tested. The enzyme from *M. globosa* showed a K_I_ in the range of 4.06–21.4 mM for most of these inhibitors, apart from carbonate, bisulfite, and peroxydisulfate. Indeed, the two human isoforms belonging to the α-CA class were well inhibited by the ‘‘metal ion poisons”, such as cyanide and azide. The behavior of MreCA is somewhat difficult to explain observing the different inhibition profile compared to MgCA, hCA I, and hCA II. However, it fortifies the thesis that the synthesis of new drugs capable of interfering selectively with MreCA and MgCA activity can avoid the inhibition of the human CAs (α-class enzymes), leading to the inactivation of the CAs encoded by the scalp microbes necessary for the integrity of the human skin.
(ii) Simple and complex anions investigated here, including iodide, nitrate, sulfamic acid, phenylboronic acid, perchlorate, osmate, perruthenate, esafluorofosfato, and trifalate, showed an interesting inhibition profile for MreCA, with K_I_ ranging between 3.9 and 9.0 mM. Most of these anions showed a similar K_I_ for the homologous enzyme MgCA, with the exception of sulfamic acid and phenylboronic acid, which are very effective inhibitors of MgCA, with K_i_ of 0.083 and 0.089 mM, respectively. Interesting, the human isoforms (hCAI and hCAII) are not well inhibited by phenyl boronic acid.
(iii) The most effective MreCA inhibitors identified in this study were bicarbonate, sulfamide, phenyl arsenic acid, stannate, tellurate, tetraborate, selenocyanate, trithiocarbonate, and diethyldithiocarbamate (K_I_ of 0.075–0.86 mM). These small molecules/anions are in fact well known to effectively inhibit many CAs belonging to all genetic families, as well as the two human isoforms (hCA I and hCA II).

## 3. Materials and Methods 

### 3.1. Cloning and Purification of MreCA

The amino acid sequence of *Malassezia restricta* corresponding to a β-CA was back-translated into the nucleotide sequence and optimized for the codon usage to increase its expression in *Escherichia coli* cells. The synthetic *M. restricta* gene, as obtained from GeneArt Company (Milan, Italy) was cloned into the expression vector to obtain the pET100D-Topo/MreCA. Competent *Escherichia coli* BL21 (DE3) codon plus cells (Agilent) were transformed with pET100D-Topo/MreCA and induced with Isopropyl β-D-1-thiogalactopyranoside (IPTG). After growth, the cells were harvested and disrupted by sonication. The cellular extract was loaded and purified onto a His-select High-Flow (HF) Nickel affinity column. After the column, the resulting enzyme was 80% pure.

### 3.2. Western Blotting

MreCA was subjected to a 12% (*w*/*v*) SDS-PAGE, followed by electrophoretic transfer to a PVDF membrane with transfer buffer (25 mM Tris, 192 mM glycine, 20% methanol) using Trans-Plot SD Cell (Bio-Rad, Hercules, CA, USA). His-Tag Western blot was carried out using the Pierce Fast Western Blot Kit (Thermo Scientific, Waltham, MA, USA). Blotted membrane had been placed in the wash blot solution Fast Western 1 Wash Buffer to remove transfer buffer. Primary Antibody Working Dilution was added to the blot and incubated for 30 min at room temperature (RT) with shaking. Afterwards, the blot was removed from the primary antibody solution and incubated for 10 min with the Fast Western Optimised HRP Reagent Working Dilution. Subsequently, the membrane was washed two times in about 20 mL of Fast Western 1 Wash Buffer. Finally, the membrane was incubated with the detection reagent working solution and incubated for 1 min at room temperature and then developed with X-ray film.

### 3.3. Determination of the Inhibition Constants

An Applied Photophysics stopped-flow instrument was used for assaying the CA catalyzed CO_2_ hydration activity [48]. Bromothymol blue (at a concentration of 0.2 mM) was used as an indicator, working at the absorbance maximum of 557 nm, with 10–20 mM TRIS (pH 8.3) as buffer and 20 mM Na_2_SO_4_ for maintaining constant ionic strength (this anion is not inhibitory and has a K_I_ > 200 mM against this enzyme), following the initial rates of the CA-catalyzed CO_2_ hydration reaction for a period of 10–100 s. The CO_2_ concentrations ranged from 1.7 to 17 mM for the determination of the kinetic parameters and inhibition constants. For each measurement, at least six traces of the initial 5–10% of the reaction were used for determining the initial velocity, working with 10-fold decreasing inhibitor concentrations ranging between 1 nM and 10–100 µM (depending on the inhibitor potency, but at least 5 points at different inhibitor concentrations were employed for determining the inhibition constants). The uncatalyzed rates were determined in the same manner and subtracted from the total observed rates. Stock solutions of inhibitor (0.1 mM) were prepared in distilled-deionized water and dilutions up to 1 nM were done thereafter with the assay buffer. Inhibitor and enzyme solutions were preincubated together for 15 min at room temperature before assay, in order to allow for the formation of the E-I complex. The inhibition constants were obtained by non-linear least-squares methods using the Cheng–Prusoff equation, and represent the mean from at least three different determinations. The human isoforms hCA I and II and the fungus MgCA were assayed in the same conditions as above, with 4-(2-hydroxyethyl)-1-piperazineethanesulfonic acid (HEPES) buffer and phenol red as an indicator [49]. All enzymes were recombinant and produced as described earlier in our laboratory [49,50,51,52,53].

## 4. Conclusions

*Malassezia restricta* is one of the micro-organisms involved in the disequilibrium between various commensals, such as *M. globosa*, *Cutibacterium acnes* (formerly named *Propionibacterium acnes*) and *Staphylococcus sp*., which contribute to dandruff and seborrheic dermatitis symptoms [39,41,42,53]. We have cloned, purified, and investigated the anion inhibition profile of the β-CA (MreCA) encoded by the *M. restricta* genome. Indeed, a full inhibition profile of MreCA, as well as the ortholog enzyme MgCA, and the possible off-targets hCA I and hCA II, was carried out with the known CA inorganic metal-complexing anions. MreCA activity, diversely from its homologous MgCA, is not readily inhibited by anions, which generally complex effectively cations (e.g., fluoride, chloride, bromide, cyanate, thiocyanate, cyanide). The most effective MreCA inhibitors were diethyldithiocarbamate, sulfamide, phenyl arsenic acid, stannate, tellurate, tetraborate, selenocyanate, trithiocarbonate, and bicarbonate. As described in the literature, the compounds investigated bind to the Zn(II) ion in the enzyme active site either by substituting the nucleophile (water molecule/hydroxide ion) as the fourth zinc ligand or by adding to the zinc coordination sphere to generate a trigonal bipyramidal geometry relative to the metal ion. The final result is a possible inactivation of the enzyme due to the degree of the structural distortion caused by the inhibitor. Thus, although CAs have a similar catalytic site, each biocatalyst has unusual architectural features, which must occur in the interaction between the protein and the anions investigated, explaining the differences in the degree of structural distortion and the different K_I_ values obtained for the four proteins studied. In conclusion, the investigation of the anion inhibition profiles of the fungal CA enzymes represents a significant starting point for designing novel metal-based antifungal treatments, which need to be further investigated using structural analysis of MrCA

## Figures and Tables

**Table 1:** Inhibition constants of anionic inhibitors against the α-CAs isoform (hCA I and hCA II) and the β-CAs from *M. globosa* and *M. restricta* (MgCA and MreCA) for the CO_2_ hydration reaction, at 20 °C and pH 8.3.

K_I_ (mM) *				
Inhibitor ^§^	hCA I ^a^	hCA II ^a^	MgCA ^a^	MreCA ^b^
F^−^	>300	>300	7.13	>50
Cl^−^	6	200	7.98	>50
Br^−^	4	63	18.6	>50
I^−^	0.3	26	8.73	8.6
CNO^−^	0.0007	0.03	6.81	>50
SCN^−^	0.2	1.6	8.39	>50
CN^−^	0.0005	0.02	7.19	>50
N_3_^−^	0.0012	1.51	45.2	>50
NO_2_^−^	8.4	63	7.56	>50
NO_3_^−^	7	35	8.13	9
HCO_3_^−^	12	85	0.59	0.86
CO_3_^2^	15	73	>100	>50
HSO_3_^−^	18	89	>100	>50
SO_4_^2−^	63	>200	19.5	>50
HS^−^	0.0006	0.04	11.9	>50
H_2_NSO_2_NH_2_	0.31	1.13	0.094	0.72
NH_2_SO_3_H	0.021	0.39	0.083	7.7
PhAsO_3_H_2_	31.7	49.2	0.09	0.83
PhB(OH)_2_	58.6	23.1	0.089	8.7
ClO_4_^−^	>200	>200	>100	9.2
SnO_3_^2−^	0.57	0.83	5.07	0.56
SeO_4_^2−^	118	112	7.41	1.7
TeO_4_^2−^	0.66	0.92	5.75	0.56
OsO_5_^2−^	0.92	0.95	6.16	8.5
P_2_O_7_^2−^	25.77	48.5	6.03	>50
V_2_O_7_^2−^	0.54	0.57	6.89	>50
B_4_O_7_^2−^	0.64	0.95	8.45	0.4
ReO_4_^−^	0.11	0.75	16.7	>50
RuO_4_^−^	0.101	0.69	8.82	7.4
S_2_O_8_^2−^	0.107	0.084	>100	>50
SeCN^−^	0.085	0.086	1.73	0.65
NH(SO_3_)_2_^2−^	0.31	0.76	21.4	>50
FSO_3_^−^	0.79	0.46	4.06	>50
CS_3_^2−^	0.0087	0.0088	1.77	0.92
Et_2_NCS_2_^−^	0.00079	0.0031	0.3	0.075
PF_6_^−^	nt	nt	6.47	3.9
CF_3_SO_3_^−^	nt	nt	2.28	4.5

* Mean from three different assays, by a stopped flow technique (errors were in the range of ± 3–5% of the reported values); § As sodium salt, except sulfamide, phenylboronic acid and phenylarsonic acid; ^a^ Data reported previously in Ref. [31]; ^b^ this work.
